# Clinical characteristics and long-term outcome of Takayasu arteritis in Iran: a multicentre study

**DOI:** 10.3906/sag-1910-19

**Published:** 2020-06-23

**Authors:** Aida MALEK MAHDAVI, Nadereh RASHTCHIZADEH, Hadise KAVANDI, Mehrzad HAJIALILO, Sousan KOLAHI, Mohammad Reza JAFARI NAKHJAVANI, Ali-asghar EBRAHIMI, Seyedmostafa SEYEDMARDANI, Mansour SALESI, Mohsen SOROUSH, Alireza KHABBAZI

**Affiliations:** 1 Connective Tissue Diseases Research Centre, Tabriz University of Medical Sciences, Tabriz Iran; 2 Rheumatology Section, Department of Internal Medicine, Urmia University of Medical Sciences, Urmia Iran; 3 Rheumatology Section, Department of Internal Medicine, Isfahan University of Medical Sciences, Isfahan Iran; 4 Rheumatology Section, Department of Internal Medicine, AJA University of Medical Sciences, Tehran Iran

**Keywords:** Takayasu arteritis, Birmingham Vasculitis Activity Score (BVAS), Indian Takayasu Clinical Activity Score (ITAS), Vasculitis Damage Index (VDI), outcome, remission, Iran

## Abstract

**Background/aim:**

This study aimed to evaluate the demographic, clinical, angiographic and prognostic characteristics of Takayasu arteritis (TA) in Iran.

**Materials and methods:**

A total of 75 patients with TA based on the American College of Rheumatology 1990 criteria for TA classification referred to the Rheumatology Centres, were followed-up from 1989 to 2019. Demographic, clinical, angiographic and prognostic characteristics were collected at baseline and last visit.

**Results:**

The mean age was 31.9 ± 9.8 years at the disease onset. Female to male ratio was 14. The median latency in diagnosis was 24 months. Pulse discrepancy in the arms, blood pressure discrepancy in the arms, limb claudication, hypertension and constitutional symptoms were the most common clinical features. The most common angiographic type at the time of diagnosis was Type I (42.7%). The most frequent arterial lesion was stenosis (89.4%). Subclavian, carotid and aortic arteries were the most commonly involved arteries. New lesions developed in 28.6% of patients during the 5.25-year follow-up. Vasculitis-induced chronic damage was observed in all patients. Disease activity decreased and vascular damage remained stable throughout the follow-up period.

**Conclusions:**

The clinical features and angiographic type of TA in Iran are different from most Asian countries. Differences in angiographic and clinical features may lead to delayed diagnosis. The issue of delay in diagnosis should create awareness among health care providers that TA is not a very rare disease in Iranians and failure to pay attention to warning symptoms may delay the diagnosis.

## 1. Introduction

Takayasu arteritis (TA) is a chronic vasculitis of unknown aetiology that affects the large and medium sized arteries, especially the aorta and its major branches. Inflammation of the arterial wall leads to thickening of the wall, stenosis, obstruction and aneurysm of the arteries [1]. These changes are responsible for the clinical manifestations of TA such as reduced blood pressure (BP) in the limbs, claudication, decreased arterial pulses, bruits over arteries, ischemic ulcerations and light-headedness [1]. Acute events like visual loss, myocardial infarction (MI), stroke or transient ischemic attacks (TIA) are other presentations of the disease [1]. Constitutional symptoms including fever, malaise, fatigue, and weight loss are nonspecific findings of TA [1].

TA occurs throughout the world and may have a varying clinical spectrum in different populations [2]. Although TA is a disease of young people that starts most commonly at the ages of 20–30, its maximum prevalence in Sweden and Italy is at 41 [2]. The ratio of women to men is 12:1 in Turkey and 9:1 in Japan, while in India it is 1:1 [2]. Considering the little information about TA in Iran, this multicentre study has been conducted on the demographic, clinical, angiographic and prognosis of TA in Iranians.

## 2. Materials and methods

### 2.1. Study group

In this cross-sectional multicentre study, the medical records of 92 patients with TA were reviewed, retrospectively. These patients were treated at the following rheumatology centres: Connective Tissue Diseases Research Centre of the Tabriz University of Medical Sciences, the rheumatology clinic in the Isfahan University of Medical Sciences, the rheumatology clinic in the Urmia University of Medical Sciences and the rheumatology clinic in the Army Hospital of Tehran. Rheumatology centres with at least 3 patients with TA were included. After excluding repeated records and patients with incomplete data, 75 patients with clinical and angiographic findings compatible with the TA met the American College of Rheumatology 1990 criteria for TA classification and complete assessment of the arterial tree in the neck, thorax, abdomen and limbs with one of the angiographic methods including computed tomography (CT) angiography, magnetic resonance (MR) angiography or conventional angiography were recruited in the study (Tables 1 and 2) [3]. In patients older than 50, the presence of giant cell arteritis and 

**Table 1:** Demographic characteristics of participants (N = 75).

Parameters	Number
Age at disease onset (years)	31.9 ± 9.8 (min: 12, max: 66)
Time to diagnosis (years)	4.28 (median 2, min: 0.1, max: 32)
Disease duration (years)	8.58 (median 6.25, min: 0.4, max: 32)
Follow-up duration (years)	5.25 (median 6, min: 0.5, max: 30)
Female: Male	14:1
Family history of TA or other rheumatic diseases	0
ACR classification criteria Age at disease onset <40 Claudication of the extremities (%) BP discrepancy in the upper extremities (%) Decreased pulsation of one or both brachial arteries (%) Bruit over subclavian artery or aorta (%) Angiographic abnormalities (%)	62 (82.7) 43 (57.3) 53 (70.7) 58 (77.3) 41 (54.7) 75 (100)

TA, Takayasu arteritis; ACR, American College of Rheumatology; BP, blood pressure.

**Table 2:** Angiographic classification of TA patients at the time of diagnosis and its associations with demographic characteristics and outcome of disease (n = 75).

Angiographic classification	Number	Age (years)	P- value*	Sex(F:M)	P- value*	Delay diagnosis (years)	P- value*	VDI at the end of follow up	P- value*	ITAS at the diagnosis	P- value*	Remission rate	P- value*
Type I (%) Type IIa (%) Type IIb (%) Type III (%) Type IV (%) Type V (%)	32 (42.7) 15 (20.0) 3 (4.0) 0 (0) 3 (4.0) 22 (29.3)	34.59 27.44 32.67 - 25.5 30.42	0.05	31:1 15:0 3:0 - 1:2 20:2	0.278	3.49 4 10 - 2 5.17	0.740	2.1 3 4 - 2.1 2.8	0.436	5.5 5.77 2 - 5.2 4.1	0.178	23 (71.9) 11 (73.3) 2 (66.7) - 2 (66.7) 16 (72.7)	0.668

*P values were calculated for angiographic type I versus other types.

polymyalgia rheumatica were excluded. Informed consents were taken from the participants in the study and the study protocol was approved by the local ethics committees.

### 2.2. Data collection

A detailed questionnaire containing demographic characteristics: age at the onset of the disease; duration of symptoms before diagnosis; duration of follow-up; clinical manifestations of the disease; disease activity in the first visit and at the end of follow-up; laboratory findings including blood count, kidney function tests, liver function tests, erythrocyte sedimentation rate (ESR), C-reactive protein (CRP); imaging findings including CT angiography, MR angiography or conventional angiography; the pattern of vascular involvement; treatment modalities including medications and revascularization procedures; morbidity and mortality were applied. Data were collected at the baseline and last visit.

### 2.3. Anatomic classification

TA was classified into 6 groups using the Hata’s angiographic classification [4] as the following: Types I, IIa, IIb, III, IV, and V. The patterns of vascular involvement were assessed by angiography, including occlusion (a total occlusion of the lumen), stenosis (a narrowing of more than 70% in the lumen) and aneurysm.

### 2.4. Pulmonary artery hypertension (PAH)

We used transthoracic echocardiography for the assessment of pulmonary artery (PA) pressure. PAH was defined as PA systolic pressure higher than 30 mmHg.

### 2.5. Assessment of disease activity and course

Disease activity was measured using national institutes of health (NIH) criteria for active disease [5], Birmingham Vasculitis Activity Score (BVAS) version 3 [6] and Indian Takayasu Clinical Activity Score (ITAS) [7]. Patients were categorized according to the disease activity in the last 6 months of the follow-up: active disease, disease in remission, or death. We defined active disease using NIH criteria. Sustained remission was applied to cases that had NIH criteria for at least 6 months. Vasculitis Damage Index (VDI) at the time of diagnosis and last visit were used for quantifying damage [8,9].

### 2.6. Statistical analysis 

For statistical analysis, we used SPSS 16 software (SPSS Inc., Chicago, IL, USA). Categorical variables were expressed as number and percentage. Continuous variables were reported as mean ± SD and for out of range data by median. Continuous variables were compared using parametric test (independent sample t-test) and/or nonparametric test (Mann–Whitney U test). Chi-squared test was used for the comparison of categorical variables. Factors correlated with delay in diagnosis of TA or remission were subjected to univariate regression analysis and were expressed as odds ratio (OR) and 95% confidence interval (95% CI). All factors with P-values less than 0.20 were assessed using multivariate regression analysis. P-values of < 0.05 were considered statistically significant.

## 3. Results

### 3.1. Demographic, clinical and laboratory characteristics

**Figure 1:**
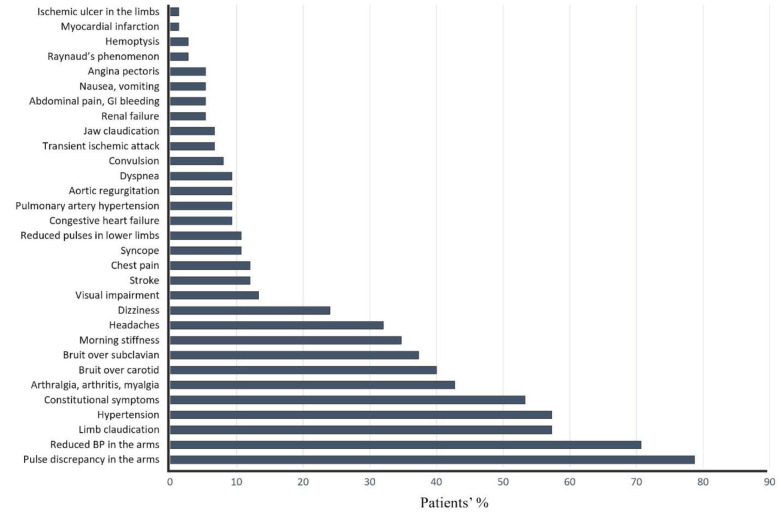
Clinical findings of participants at diagnosis (N = 75). GI, gastrointestinal

**Figure 2:**
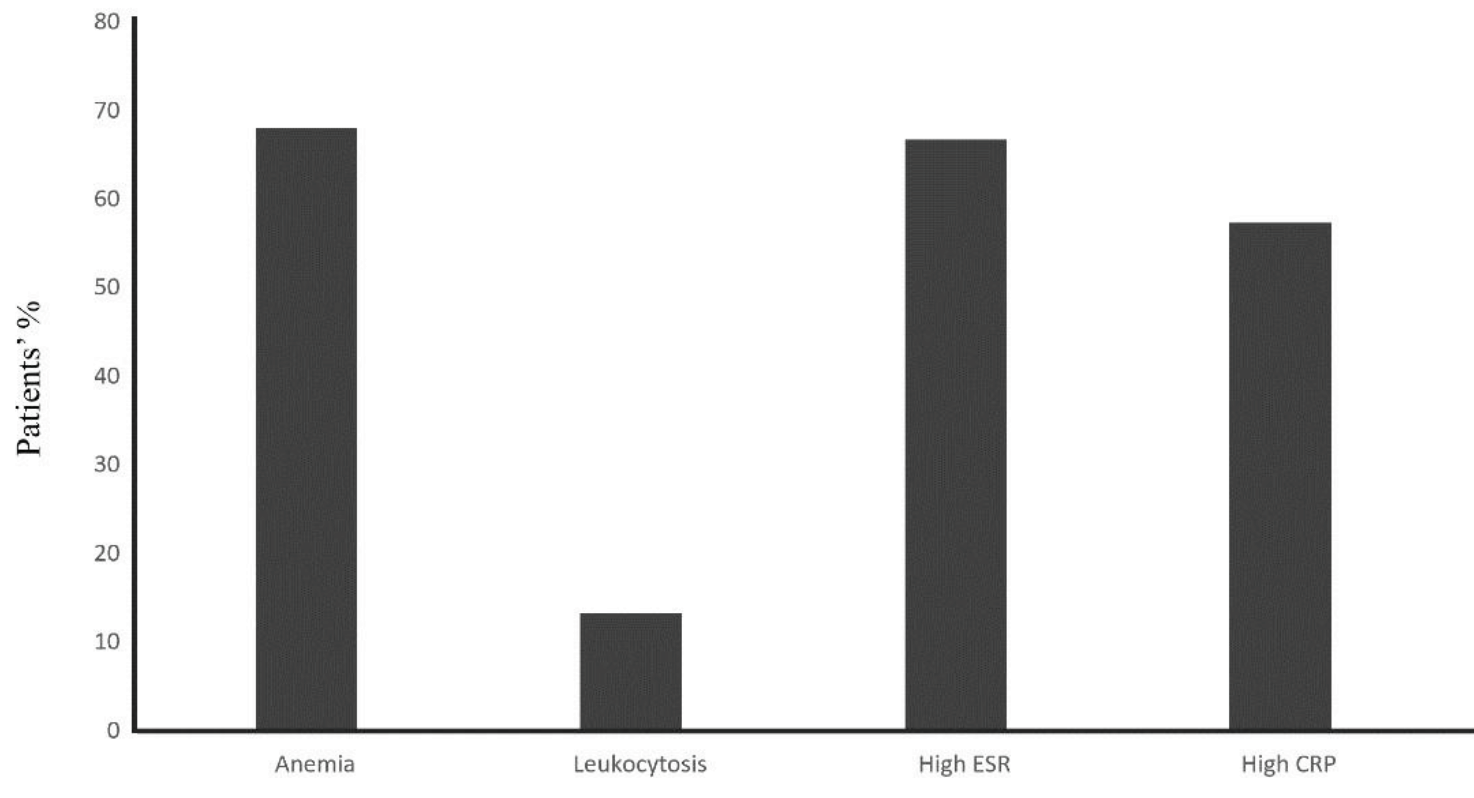
Laboratory findings of participants at diagnosis (N = 75). ESR, erythrocyte sedimentation rate; CRP, C-reactive protein.

The study included 75 patients with TA followed between 1989 and 2019. Demographic characteristics of patients are summarized in Table 1. Mean duration of follow-up was 5.25 (min: 0.5, max: 30) years. Female to male ratio was 14. The onset of the disease in 13 (17.3%) patients was after 40 years of age. The median latency in diagnosis was 24 months. Using regression analysis, we were not able to identify variables as predictors of latency in diagnosis (Table 3). The clinical features of the studied patients are shown in Figure 1. Pulse discrepancy in the arms, BP discrepancy in the arms, limb claudication, hypertension and constitutional symptoms were the most common features. Laboratory findings are evident in Figure 2. In 54 (72%) patients at least one of the laboratory tests (ESR, CRP, and CBC) was abnormal.

**Table 3:** Factors associated with delay of >2 years in diagnosis of TA.

Parameters	Univariate analysis		Multivariate analysis	
	OR (95% CI)	P-value	OR (95% CI)	P-value
Age at the diagnosis of TA > 30	0.26 (0.08–0.86)	0.028	0.34 (0.09–1.23)	0.100
Female sex	2.28 (0.22–13.74)	0.489		
Clinical manifestations				
Constitutional symptoms, myalgia, arthralgia, arthritis	1.47 (0.43–4.99)	0.557		
Upper limb claudication or BP discrepancy or pulse discrepancy	0.72 (0.17–3.10)	0.659		
Bruit over subclavian artery	1.07 (0.33–3.48)	0.908		
Bruit over carotid artery	3.27 (0.95–11.24)	0.060	2.31 (0.59–9.12)	0.232
CNS symptoms	2.23 (0.67–7.40)	0.192	1.25 (0.32–4.91)	0.753
Hypertension	2.03 (0.64–6.49)	0.232		
PAH	1.05 (0.14–8.13)	0.965		
Chest pain, MI and dyspnoea	2.13 (0.58–7.85)	0.254		
High ESR or CRP	0.95 (0.28–3.24)	0.938		
Angiographic type 1 versus others	0.68 (0.23–1.99)	0.481		
Angiographic type 5 versus others	0.85 (0.63–1.15)	0.292		

TA, Takayasu arteritis; OR, odds ratio, CI, confidence interval; BP, blood pressure; CNS, central nervous system; PAH, pulmonary artery hypertension; MI, myocardial infarction; ESR, erythrocyte sedimentation rate; CRP, C-reactive protein.

### 3.2. Angiographic findings

CT angiography was performed in 73 (97.3%) patients. In 2 patients, MR angiography was performed for imaging. Patients were classified according to the angiographic findings at the time of diagnosis (Table 2). Type I was the most common type. No case of Type III was observed. Arterial stenosis was the most frequent arterial lesion (89.4%). Dilatation or aneurysm (11.1%) and finally occlusion (7.2%) were less frequent. Patients with Type I were significantly older than the patients in other types (Table 2). No significant association was observed between other demographic characteristics, the outcome and angiographic types of the disease (Table 2). The distribution of arterial involvement is shown in Table 4. Subclavian and carotid arteries and aortic arc were the most commonly involved arteries. During the follow-up imaging, studies were repeated in 28 patients. New lesions developed in 8 patients (28.6%).

**Table 4:** Anatomical distribution and pattern of arterial lesions at the time

Anatomical distribution	Any lesion	Stenosis	Obstruction	Aneurysm
Subclavian artery (%)	63 (84.0)	52 (82.5)	10 (15.9)	4 (6.3)
Carotid artery (%)	36 (48.0)	33 (91.7)	1 (2.8)	4 (11.1)
Aortic arc (%)	26 (34.7)	22 (84.6)	0	5 (19.2)
Renal artery (%)	17 (22.7)	16 (94.1)	1 (5.9)	0
Abdominal aorta (%)	12 (16.0)	11 (91.7)	1 (8.3)	1 (8.3)
Brachial artery (%)	10 (13.3)	10 (100)	0	1 (1.4)
Thoracic aorta (%)	8 (10.7)	6 (75.0)	0	4 (50)
Vertebral artery (%)	10 (13.3)	9 (10.0)	1 (1.0)	0
Pulmonary artery (%)	6 (8.0)	6 (100)	0	2 (25)
Axillary artery (%)	5 (6.7)	5 (100)	1 (20.0)	0
Ascending aorta (%)	5 (6.7)	1 (25.0)	0	4 (75.0)
Mesenteric artery (%)	5 (6.7)	5 (100)	0	0
Celiac artery (%)	4 (5.3)	4 (100)	0	0
Iliac artery (%)	4 (5.3)	4 (100)	0	0
Cerebral artery (%)	4 (5.3)	4 (100)	0	0
Coronary artery (%)	1 (1.3)	1 (100)	1 (100)	0

### 3.3. Disease activity, treatment and outcome

Disease was active in 62 (82.7%) patients at the time of diagnosis. Treatment with prednisolone was started in 66 (88%) patients. In 47 (62.7%) patients at least one disease modifying antirheumatic drug (DMARD) was added to the treatment regime (Table 5). During the follow-up period disease activity (ITAS and BVAS) decreased, and vascular damage remained stable (Table 5). In 54 (72%) patients the disease was in sustained remission at the end of the follow-up. Apart from the age of the patients at disease presentation, less latency in diagnosis and higher ITAS at the baseline, no significant differences were observed between TA patients with disease in remission and active disease (Table 6). However, using regression analysis, we were not able to identify variables as predictors of remission in TA patients (Table 7). Five pregnancies were recorded during the follow-up period, all of which lead to live new-borns. Disease activity decreased in 2 patients, however no change happened in 3 patients.

**Table 5:** Therapies and outcomes of TA patients (N = 75).

Parameters	Number
Medications	
Prednisolone (%)	66 (88)
Methotrexate (%)	36 (48)
Azathioprine (%)	16 (21.3)
Cyclosporine (%)	2 (2.7)
Cyclophosphamide (%)	5 (6.7)
Mycophenolate mofetil (%)	5 (6.7)
Biologics (%)	9 (12)
DMARDs number 0 (%) 1 (%) 2 (%) ≥3 (%)	28 (37.3) 37 (49.3) 8 (10.7) 2 (2.6)
Prednisolone dose at the baseline (mg/d)	34.26 ± 17.9
Prednisolone dose at the end of follow-up (mg/d)	11.1 ± 4.8
ITAS at the baseline	5.3 (median = 5.1, min = 0, max = 15)
ITAS at the end of follow-up	1.5 (median = 1, min = 0, max = 5)
BVAS at the baseline	3.8 (median = 3, min = 0, max = 12)
BVAS at the end of follow-up	2.1 (median 3, min = 0, max = 4)
VDI at the baseline	2.5 (median 2, min = 1, max = 4)
VDI at the end of follow-up	2.6 (median 2.5, min = 1, max = 4)
Disease course at the end of follow-up In remission (%) Active (%) Death (%)	54 (72.0) 17 (22.7) 4 (5.3)
Open surgery (%)	8 (10.7)
Angioplasty (%)	8 (10.7)
Morbidity (%)	41 (55.4)
Job loss (%)	7 (9.5)

TA, Takayasu’s arteritis; DMARDs, disease-modifying antirheumatic drugs; BVAS, Birmingham Vasculitis Activity Score;

**Table 6:** Comparison of demographic data, clinical characteristics, angiographic classification, and treatment regimen between TA patients with disease in remission and active disease.

Parameters	TA in remission(N = 54)	Active TA (N = 21)	P-value
Age at disease presentation (years)	26.3 ± 9.2	32.9 ± 10.7	0.042
Female (%)	48 (88.9)	18 (85.7)	0.640
Delay in diagnosis (years)	4.3 (median = 2)	1.8 (median = 1.7)	0.035
ESR (mm/h)	51.2 (median = 50)	49.4 (median = 37)	0.347
Angiographic type 1 versus others	26 (48.2)	6 (28.6)	0.459
Angiographic type 5 versus others	17 (31.5)	5 (23.8)	0.765
Prednisolone dose at the cohort entry (mg/d)	36.7 (median = 30)	34.1 (median = 30)	0.786
Treatment with DMARDs (%)	32 (59.3)	12 (57.1)	0.713
ITAS at the baseline	5.1 (median = 5)	5 (median = 5.5)	0.651
BVAS at the baseline	1.7 (median = 1)	5 (median = 4)	0.026
Surgery or intervention (%)	10 (18.5)	3 (14.3)	0.597

TA, Takayasu arteritis; DMARDs, disease-modifying antirheumatic drugs; BVAS, Birmingham Vasculitis Activity Score; ITAS, Indian Takayasu Activity Score.

**Table 7:** Factors associated with remission in TA.

Parameters	Univariate analysis		Multivariate analysis	
	OR (95% CI)	P-value	OR (95% CI)	P-value
Age at the diagnosis of TA > 30	2.08 (0.49–8.87)	0.124	5.79 (0.68–0.87)	0.204
Female sex	0.88 (0.21–7.43)	0.915		
Delay in diagnosis >2 years	2.73 (0.87–9.05)	0.091	1.84 (0.53–10.21)	0.165
Angiographic type 1 versus others	1.44 (0.35–5.95)	0.613		
Angiographic type 5 versus others	1.11 (0.98–1.47)	0.160	1.01 (0.98–1.09)	0.191
Severe activity according NIH criteria at the entry of cohort	0.47 (0.19–1.13)	0.092	0.34 (0.06–1.19)	0.067
Prednisolone dose at the entry of cohort > 30 mg/d	0.71 (0.18–2.73)	0.613		
Treatment with DMARDs	0.86 (0.20–3.64)	0.834		

TA, Takayasu arteritis; OR, odds ratio, CI, confidence interval; NIH, National Institute of Health; DMARDs, disease-modifying antirheumatic drugs.

Fourteen patients (18.7%) experienced a total of 16 (21.3%) vascular interventions. A percutaneous transluminal angioplasty in 8 (10.7%), aortic valve replacement in 6 (8%), and bypass in 2 (26.7%) of these patients were performed. The indications for vascular interventions were aortic insufficiency (10.7%), renal vascular hypertension (5.3%), limb claudication (2.7%), cerebral ischemia (1.3%), and abdominal ischemia (1.3%).

At the 5.25-year follow-up, stroke in 8 (10.7%), PAH in 7 (9.3%), congestive heart failure (CHF) in 6 (8%), renal failure in 4 (5.3%), MI in 1 (0.8%), and deaths in 4 (5.3%) patients occurred (Figure 1). The causes of death were CHF (2.7%), stroke (1.3%) and septic shock (1.3%). The calculated 5-year and 10-year survival rates were 94.9% and 89.9%, respectively, in this cohort.

## 4. Discussion

In this study, we presented demographic, clinical and imaging features, disease activity and outcomes of 75 Iranian patients with TA. Clinical manifestations, angiographic findings and outcomes in patients with TA vary in different geographic regions. We compared demographic characteristics, clinical features and outcomes of patients in our study with studies from other countries (Table 8) [4,10–22].

**Table 8:** Comparison of demographic, clinical and outcome data of TA from literature.

Study	Number of studied patients	Country	Mean age	Female to male ratio	The mean lag time from symptom onset to diagnosis (months)	Most common involved arteries	Mostcommon angiographic type	Active disease atdiagnosis	Adding DMARDs to treatment regimen	Surgeries and vascular interventions, and outcomes	Sustained remission	Survival rates
Hata et al. 1996 [4]	80	Japan	37	25.7	-	Carotid, subclavian	Type V (54%)	-	-	-	-	-
Hata et al. 1996 [4]	102	India	28	1.7	-	Renal, subclavian	Type V (55%)	-	-	-	-	-
Park et al. 2005 [10]	108	Korea	29	5.4	14	Subclavian (34%), renal (25%)	Type I (36%)	84%	36%	-	75%	87.2% at the tenth year
Yang et al. 2014 [11]	556	China	29	3.8	91	Left subclavian artery (49%), left common carotid artery (31%)	Type III(37.8%)	23%	4%	Surgery in 35%	66%	94% at the fifth year
Li et al. 2017 [12]	411	China	23	3.8	21	Subclavian (80%), carotid (79%)	Type V (61%)	-	-	Surgery in 13% and intervention in 11%	-	-
Goel et al. 2017 [13]	251	India	29	4.6	50	-	Type V (54%)	5.2%	94%	Intervention in 72%, surgery in 3%	46%	98% at the tenth year
Bicakcigil et al. 2009 [14]	248	Turkey	30	8.2	34	Subclavian(76%), carotid (52%)	Type V (51%)	93%	84%	Surgery or intervention in 26%	71%	-
Karageorgaki et al. 2009 [15]	42	Greece	31	7.4	24	Subclavian (97%), carotid (91%)	Type V (70%)	-	82%	Surgery in 43% and intervention in 21%	83% (partial in 55% and complete in 28%)	-
Vanoli et al. 2005 [16]	104	Italy	29	7	42	Subclavian, carotid	-	54%	-	Surgery in 50% and intervention in 54%	69%	-
Petrovic-Rackov et al. 2009 [17]	16	Serbia	30	All were female	24	Subclavian (88%), carotid (37%)	Type I (50%)	-	69%	Surgery or intervention in 44%;	94%	-
Gudbrandsson et al. 2016 [18]	77	Norway	32	11.5	37	Subclavian, carotid	Type I (47%)	-	-	-	-	-
Arnaud et al. 2010 [19]	82	France	33	4.8	-	Carotid, subclavian	Type V (48%)	-	53%	Surgery or intervention in 49%	-	100% at 5 years and 95.0% at 10 years
Schmidt et al. 2013 [20]	126	USA	29	10	17.5	Subclavian (66%), carotid (55%)	Types V (57%)	-	66%	Surgery or intervention in 55%; with surgical mortality of 3%	71%	97% at the tenth year
Soto et al. 2007 [21]	94	Mexica	26	5.7	-	Subclavian(65%), carotid (48%)	Type V (69%)	32%	-	Surgery in 24% and intervention in 7%	-	73% at the tenth year
Sato et al. 1998 [22]	73	Brazil	27	5	-	-	Type V (57%)	92%	33%	Surgery or intervention in 11%	-	93% at the fifth year
Present study	75	Iran	32	14	51	Subclavian (84%), carotid (46%)	Type I (43%)	83%	63%	Surgery in 11% and intervention in 11%	72%	90% at the tenth year

TA, Takayasu’s arteritis; Nasu’s classification of an angiogram in Takayasu arteritis.

### 4.1. Demographic characteristics

In studied patients, the mean age at onset of TA was 32 years, similar to the age of the onset in Asia, Europe, and North and South America (Table 8). Female predominance was highest in our patients after Japan (Table 8). TA was introduced as a disease present in patients below 40. This study showed that TA in 17% of Iranian patients started after the age 40, whilst this figure was 14% in Turkish, 17.5% in Italian, 26% in Norwegian, and 32.0% in French patients [11,14,16,18,19].

There was a 51-months delay between disease presentation and diagnosis of TA in Iran. The interval from the initial symptoms of TA until the time of the diagnosis in other series was 14–91 months (Table 8). None of the demographic, clinical and laboratory parameters were predictors of delay in diagnosis in the studied patients. In a report by Karageorgaki et al., delay in diagnosis of TA was 24 months and fatigue at the time of diagnosis was associated with an increased risk for delay in diagnosis. Vanoli et al. reported the age < 15 years and an ESR < 30 at disease onset as risk factors associated with a diagnosis delay ≥ 2 years [16].

### 4.2. Angiographic findings

The most common angiographic type in the studied patients was Type I, whereas in countries with high prevalence of TA (Japan, India and China) and in most European countries and USA, the most common type was V (Table 8). The distribution of the vascular stenosis in our study is consistent with those in other studies, which means that the most commonly involved arteries are subclavian and carotid.

### 4.3. Treatment and outcome

Glucocorticoids (GCs) are the mainstay of TA treatment. However, in 63% of our patients DMARDs were used in order to get remission or decreasing GC dose. The figure varies from 4%–98% in different countries (Table 8). Although remission occurred in the majority of TA patients, 52% of them experienced at least one of the morbidities of the disease and more than 20% of the patients underwent interventional or surgical procedures. During 5.2 years of follow-up, disease stayed in remission in 72% of the patients. In our study, the survival rate was 90%, 10 years after the diagnosis. Studies from other countries reported a rate ranged from 46%–94% for remission and 73%–98% for 10-year survival (Table 8).

In our study, younger age of patients at disease presentation and more diagnosis delay were associated with sustained remission. The association between diagnosis delay and remission rate may be related to a milder nature of the disease compared to the patients who were diagnosed with more delay. In Schmidt et al. study no association was found between sex, ESR, CRP level, or angiographic classiﬁcation with risk of relapse or remission [20]. Older age at diagnosis was associated with an increased likelihood of sustained remission (hazard ratio of 1.89 per 10-year increase in age) [20]. We did not find any association between angiographic type and remission rate. However, in Goel et al. study, low CRP and angiographic type 4 were associated with a low remission rate in TA [13].

Our study had some important limitations: i) retrospective design of the study; ii) heterogeneous treatment regimens and the treatment durations present a risk of bias when evaluating the effects of the therapy on progression of vascular lesions; iii) existing serious complications before starting treatment in almost all the patients.

In conclusion, this multicentre study provides novel data on the demographic and clinical characteristics, angiographic findings, treatment and outcome of TA in Iran. The clinical features and angiographic type of TA in Iran are different from most Asian countries and similar to the North European countries. Angiographic Type 1 is the most common type in Iranians. The delay in diagnosis is high in Iran. Differences in angiographic and clinical features may lead to delayed diagnosis. The issue of delay in diagnosis should create awareness among health care providers that TA is not a very rare disease in Iranians and failure to pay attention to warning symptoms may delay the diagnosis. Earlier diagnosis and treatment of TA may improve prognosis of this disease.

## Acknowledgement

We thank all the patients for their participation in this research.

## Informed Consent

The study protocol was approved by the Ethics Committee of Tabriz University of Medical Sciences (Ethic code 5/D/486340). Written informed consent was obtained from all participants.
